# Deep learning vs. atlas-based models for fast auto-segmentation of the masticatory muscles on head and neck CT images

**DOI:** 10.1186/s13014-020-01617-0

**Published:** 2020-07-20

**Authors:** Wen Chen, Yimin Li, Brandon A. Dyer, Xue Feng, Shyam Rao, Stanley H. Benedict, Quan Chen, Yi Rong

**Affiliations:** 1 Department of Radiation Oncology, Xiangya Hospital, Central South University, Changsha, China; 2 Department of Radiation Oncology, University of California Davis Medical Center, 4501 X Street, Suite 0152, Sacramento, California 95817 USA; 3 Department of Radiation Oncology, Xiamen Cancer Center, The First Affiliated Hospital of Xiamen University, Xiamen, Fujian China; 4 Department of Radiation Oncology, University of Washington, Seattle, WA USA; 5 Carina Medical LLC, 145 Graham Ave, A168, Lexington, KY 40536 USA; 6 Department of Radiation Oncology, Markey Cancer Center, University of Kentucky, RM CC063, 800 Rose St, Lexington, KY 40536 USA

**Keywords:** Deep learning model, Masticatory muscles, Auto-segmentation

## Abstract

**Background:**

Impaired function of masticatory muscles will lead to trismus. Routine delineation of these muscles during planning may improve dose tracking and facilitate dose reduction resulting in decreased radiation-related trismus. This study aimed to compare a deep learning model with a commercial atlas-based model for fast auto-segmentation of the masticatory muscles on head and neck computed tomography (CT) images.

**Material and methods:**

Paired masseter (M), temporalis (T), medial and lateral pterygoid (MP, LP) muscles were manually segmented on 56 CT images. CT images were randomly divided into training (*n* = 27) and validation (*n* = 29) cohorts. Two methods were used for automatic delineation of masticatory muscles (MMs): Deep learning auto-segmentation (DLAS) and atlas-based auto-segmentation (ABAS). The automatic algorithms were evaluated using Dice similarity coefficient (DSC), recall, precision, Hausdorff distance (HD), HD95, and mean surface distance (MSD). A consolidated score was calculated by normalizing the metrics against interobserver variability and averaging over all patients. Differences in dose (∆Dose) to MMs for DLAS and ABAS segmentations were assessed. A paired t-test was used to compare the geometric and dosimetric difference between DLAS and ABAS methods.

**Results:**

DLAS outperformed ABAS in delineating all MMs (*p* < 0.05). The DLAS mean DSC for M, T, MP, and LP ranged from 0.83 ± 0.03 to 0.89 ± 0.02, the ABAS mean DSC ranged from 0.79 ± 0.05 to 0.85 ± 0.04. The mean value for recall, HD, HD95, MSD also improved with DLAS for auto-segmentation. Interobserver variation revealed the highest variability in DSC and MSD for both T and MP, and the highest scores were achieved for T by both automatic algorithms. With few exceptions, the mean ∆D98%, ∆D95%, ∆D50%, and ∆D2% for all structures were below 10% for DLAS and ABAS and had no detectable statistical difference (*P* > 0.05). DLAS based contours had dose endpoints more closely matched with that of the manually segmented when compared with ABAS.

**Conclusions:**

DLAS auto-segmentation of masticatory muscles for the head and neck radiotherapy had improved segmentation accuracy compared with ABAS with no qualitative difference in dosimetric endpoints compared to manually segmented contours.

## Introduction

Advances in radiotherapy techniques, such as intensity modulated radiotherapy, have improved dose conformity to radiation targets, resulting in decreased dose to adjacent organs at risk (OARs) [1, 2]. This has resulted in improved locoregional tumor control, as well as reduced incidence of late normal tissue side effects. As a result of these technological advancements, accurate and consistent delineation of tumor and OAR structures is imperative for optimal radiation planning. However, it is a labor-intensive process to manually delineate every structure. Furthermore, given the complexity of head and neck cancer (HNC) anatomy intra- and inter-observer variations in manual segmentations are common and due to the substantial time required, some OARs may not be routinely contoured [3–6].

The development of computational tools to automatically generate OAR contours can reduce the time and effort required for HNC contouring and plan development, as well as inter-observer contour variations. Specifically, organ auto-segmentation has been extensively studied [7–10] using both CT and MR image datasets [11, 12]. One approach, atlas-based auto-segmentation (ABAS) [13, 14], is a traditional method for organ contouring and various factors can affect segmentation performance. These include the size of the dataset used to create the atlas, approaches for image registration, and approaches for label fusion. Because the atlas size is fixed, the main limitation for ABAS is the ability to overcome variations in patient anatomy. In recent years, deep learning-based methods [15, 16] have shown great success for biomedical image segmentation and have been introduced to the field of head and neck anatomy segmentation. However, the literature is limited in assessing masticatory muscles (MMs) auto-segmentation [17, 18], which may be due to the lack of delineation guidelines for MMs.

Trismus, pain or difficulty with opening the mouth, is a common radiation-induced toxicity [19]. It may result in poor dental hygiene, impaired chewing, malnutrition and psychological difficulties which will eventually lead to impacts on patients’ health-related quality of life [20, 21]. Risk factors including surgery, tumor location, and high radiotherapy dosage contribute to trismus. It was observed in 35–55% of advanced oropharyngeal cancers patients treated with radiotherapy [22, 23]. Movement of the mandibular is controlled by the temporo-mandibular joint and the synergistic actions of the paired MMs consisting of the masseter (M), temporalis (T), medial pterygoid (MP) and lateral pterygoid (LP) muscles. When the MMs are within the field of radiation, fibrosis may lead to trismus, reducing the range of movement. Therefore, to reduce HNC toxicities and improve quality-of-life, it is necessary to optimize radiation dose to the target and sparing the MMs. Several dosimetric studies [22, 24, 25] investigated the relationship between radiotherapy dose to MMs and trismus. In a study of 421 cases, Rao et al. found that limiting the high dose volume of the ipsilateral MP to V68Gy < 10 cm^3^ reduced the incidence of trismus [22]. However, no standardized MM OAR definition exists, nor dose threshold for the MMs.

Previous studies evaluated the use of auto-segmentation to improve interobserver variability in contouring MMs [18]. However, to the best of our knowledge, this is the first paper to evaluate a deep learning model for auto-segmentation of MMs. This study aimed to evaluate the feasibility and performance of deep learning auto-segmentation (DLAS) compared with ABAS for paired MM auto-segmentation in terms of geometry and dosimetry accuracy. Furthermore, the performance of automatic algorithms with respect to the interobserver variability in manual contouring was evaluated.

## Materials and methods

### Imaging data

In this study, 56 head and neck (HNC) patients between 2016 and 2018 were retrospectively selected under institutional review board approval. A variety of primary head and neck disease sites for patients receiving definitive and adjuvant radiotherapy were included. The treatment was delivered via Volumetric Modulated Arc Therapy (VMAT) with the prescription dose for high-risk regions ranging from 60Gy to 70Gy in 30 to 35 fractions. Patients characteristics are shown in Table 1. All patients were staged according to the 8th AJCC staging system [26].

**Table 1:** Patients characteristics

Characteristics	Training group (*n* = 27)	Validation group(*n* = 29)
Primary site		
Oropharynx	16 (59.3%)	20 (69.0%)
Larynx	2 (7.4%)	4 (13.8%)
Nasopharynx and Sinonasal	4 (14.8%)	2 (6.9%)
Other sites	5 (18.5%)	3 (10.3%)
Stage		
I	3 (11.1%)	2 (6.9%)
II	3 (11.1%)	3 (10.3%)
III	5 (18.5%)	6 (20.7%)
IV	16 (59.3%)	17 (58.6%)
N/X	0 (0%)	1 (3.5%)
Primary Tumor Surgery		
Yes	15	17
No	12	12

The four paired masticatory muscles, masseter (M), temporalis (T) and medial/lateral pterygoids (MP, LP) muscles were contoured on a simulation CT scan. All the contours were delineated by the same HNC radiation oncologist. The contours were then reviewed and modified if necessary by a senior expert oncologist. All muscles were delineated using the soft tissue window and following the guidelines by Rao *et al* [22]. The CT images and segmented contours were extracted as DICOM files and imported to the deep learning-based contouring software and the commercial software available on the RayStation Treatment Planning (RaySearch Laboratories AB, Stockholm, Sweden) for further testing.

### Deep learning model for image segmentation

The deep learning based contouring software (INTContour, Carina Medical LLC, Lexington, KY) employs 3D U-Net structure [27] for organ segmentation. The algorithm has achieved good performance in 2017 AAPM thoracic challenge [28] and 2019 RT-MAC challenge [29]. The original CT was resampled to have the same spatial resolution, matrix size and field of view. Two 3D U-Nets with and without dilated convolutions were trained and the output from both networks was averaged. Training and testing augmentations such as random translation, rotation, scaling and left-right flipping were used to improve the model performance. The summation of the weighted cross entropy and soft Dice loss was used as the loss function. A detailed description of the segmentation method was previously published [30, 31]. From the initial dataset of 56 patients, 27 were randomly selected for training and validation during the training process. After the model was trained, the remaining 29 patients were used for testing the performance. No model re-tuning and re-testing was performed.

### Multi-atlas-based auto segmentation

Datasets were imported in RayStation treatment planning system version 9A. Multi-atlas-based auto-segmentation algorithm (ABAS) [32] was used to generate contours. The same CT images and contour sets in the training cohort (*n* = 27) for deep learning model creation were used to build the atlas. For the new imaging dataset, multiple atlas contours were first rigidly registered to the new image to identify the best matching, which was then deformed and registered to the new CT image as the new automatic generated segmentation set. An ANAtomically Constrained Deformation Algorithm (ANACONDA) was used for image deformation in the process of ABAS in Raystation [33]. This algorithm uses both intensity-based and anatomic information-based approaches to calculate deformation vectors to achieve the best match between images, the rest of 29 datasets was used for ABAS validation.

### Interobserver variability

To assess the automatic algorithms with respect to the interobserver variability in manual contouring, five head and neck CT image sets were randomly selected for MM OAR segmentation by three physicians according to the afore-mentioned MM contouring guidelines. Paired MMs were segmented and interobserver variability was assessed by pairwise comparison of MM manual contours.

### Evaluation of geometric accuracy

Dice similarity coefficient (DSC), recall, precision, Hausdorff distance (HD), HD95, and Mean surface distance (MSD) were calculated to evaluate DLAS and ABAS auto-segmentation of MM contours compared with the manually segmented gold standard. Interobserver variability was also evaluated using the same metrics. The DSC, recall, and precision are measures of overlap of two volumes (Vx and Vy) and is defined as:

$$ \mathrm{DSC}=\frac{2\left|\mathrm{Vx}\cap \mathrm{Vy}\right|}{\left|\mathrm{Vx}\right|+\left|\mathrm{Vy}\right|},\mathrm{Precision}=\frac{\left|\mathrm{Vx}\cap \mathrm{Vy}\right|}{\left|\mathrm{Vy}\right|},\mathrm{Recall}=\frac{\left|\mathrm{Vx}\cap \mathrm{Vy}\right|}{\left|\mathrm{Vx}\right|} $$

Vx is the reference contour, Vy is the contour to be evaluated. The range of the above three metrics are [0, 1], with 1 being the best value, and 0 being the worst.

The HD is the maximum distance of a point in one contour to the closest point of the other contour, while HD95 is to measure the 95% distance of all point in one contour to the other, “x” and “y” denotes the points on contour X and contour Y. It defines as:

$$ {\mathrm{d}}_{\mathrm{HD}}=\max \left(\underset{x\in X}{\min }d(x),\underset{y\in Y}{\min }d(y)\right) $$

$$ {\overrightarrow{d}}_{HD95}\left(X,Y\right)={k}_{95}\left({}_{y\in Y}^{\min \kern0.5em d\left(x,y\right)}\right),{d}_{HD95}\left(X,Y\right)=\frac{{\overrightarrow{d}}_{HD95}\left(X,Y\right)+{\overrightarrow{d}}_{HD95\left(Y,X\right)}}{2} $$

The directed mean surface distance is the average distance of a point in contour X to its nearest point in contour Y. which defines as:

$$ {\overrightarrow{d}}_{avg}\left(X,Y\right)=\frac{1}{\left|x\right|}\sum \limits_{x\varepsilon X}\underset{y\in Y}{\min}\mathrm{d}\left(x,y\right) $$

The mean surface distance (MSD) is the average of the two directed mean surface distances:

$$ {d}_{avg}\left(X,Y\right)=\frac{{\overrightarrow{d}}_{avg}\left(X,Y\right)+{\overrightarrow{d}}_{avg}\left(Y,X\right)}{2} $$

The above distance measures have a unit of cm in this study, with 0 as the most ideal value.

In this study, considering the range variation in different metrics mentioned above, a score measure was used normalizing to the interobserver variability values generated from the above five cases contoured by three physicians. It defines as:

$$ Score=\mathit{\operatorname{Max}}\left(\left(50+\frac{\left(T-R\right)}{\left(P-R\right)}\times 50\right),0\right) $$

where T presents measures of the test contours, P presents the perfect measure (i.e. DSC = 1, MSD/HD95 = 0), and R presents the reference measure for the structure. The mean score from the inter-observer study was used as the reference measure. A score of 100 indicates the highest value for all metrics, 50 is equivalent to the mean interobserver reference, and 0 indicates below the reference by the amount higher than the difference between the highest value and the reference. The generalized scores for each structure were calculated by averaging the normalized scores over three metrics (DSC, HD95, MSD) among all patients.

In addition, we counted the cases where auto-segmentation perform worse than manual segmentation by compared with the mean DSC of inter-observer variation for each muscle and calculated the rates for both DLAS and ABAS methods.

### Evaluation of dosimetric impact of variation in contouring

Dose-volume histograms (DVH) and dose statistics were computed for auto-segmented contours and manual contours (reference) using the original planned dose distribution. Pairwise comparison was performed for these three sets of statistics. Dosimetric metrics of manual contours and auto-segmented contours for each MM were assessed. The dose-related effects strictly due to the contouring variation were quantified by dose metric variation. The dose differences (∆dose) of D98% (the minimum absorbed dose, Gy), D95% (the prescribed dose, Gy), D50% (the median absorbed dose, Gy), and D2% (the maximum absorbed dose, Gy) for each muscle were calculated. The dose difference between manual and auto-segmented contours was calculated as:

$$ \Delta  dose=\mid \frac{\left({dose}_{MS}-{dose}_{DLAS/ ABAS}\right)}{dose_{MS}}\mid $$

with *dose*_*MS*_ equals to the dose of manual segmentation contours, and *dose*_*DLAS*/*ABAS*_ equals to the dose of auto-segmentation contours using either DLAS or ABAS.

### Statistical analysis

Analysis was performed using GraphPad Prism version 6 (Graph pad software) and SPSS software version 24.0 (SPSS Inc., Chicago, IL, USA). A paired t-test was used to compare the difference value of DSC, recall, precision, HD, HD95, MSD, overall scores and ∆dose between DLAS and ABAS. Chi-Square test was used to compare the rates of worse cases between DLAS and ABAS. Statistical significance was defined as *p* < 0.05.

## Results

### Variation in contouring

**Fig. 1:**
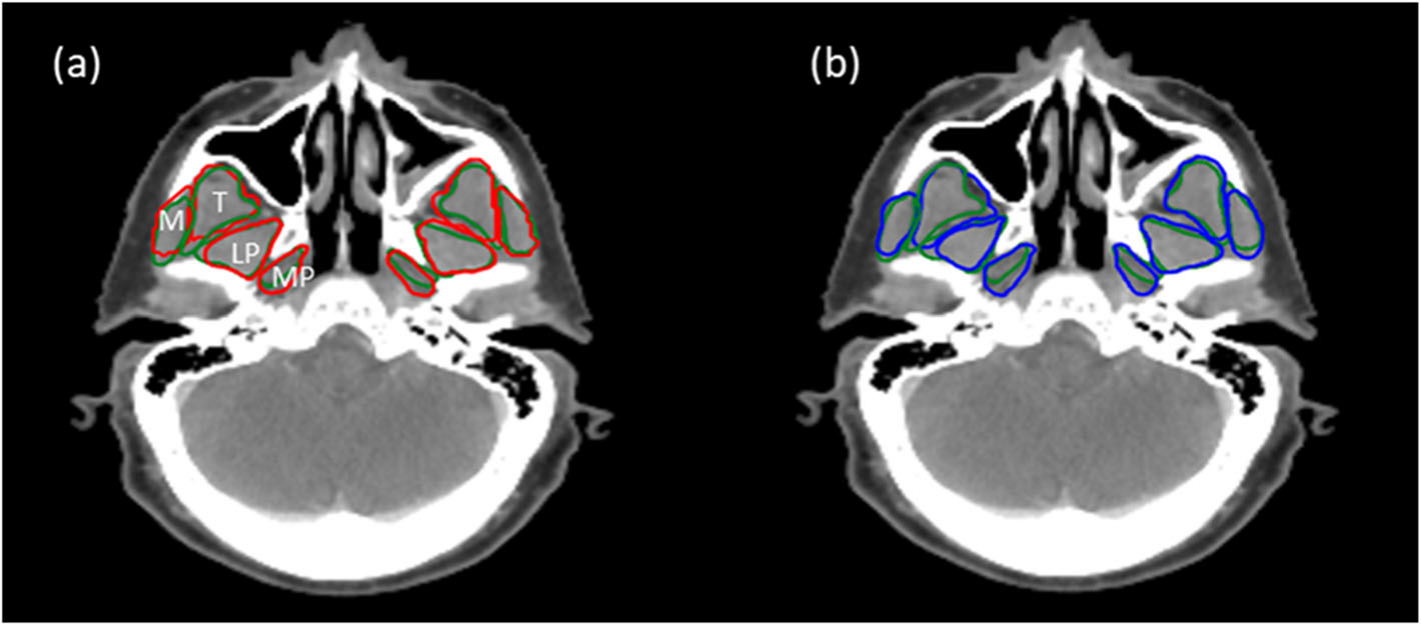
Transverse view of different contours for one presentative patient. **a** manual contours (green lines, reference standard) vs. DLAS (red lines), (**b**) manual contours (green lines) vs. ABAS (blue lines)

In all cases, both DLAS and ABAS can segment the muscles with an overall good representation. Figure 1 shows an example of the DLAS, ABAS, and manual contours. Contour variability was greatest for MP structures.

**Fig. 2:**
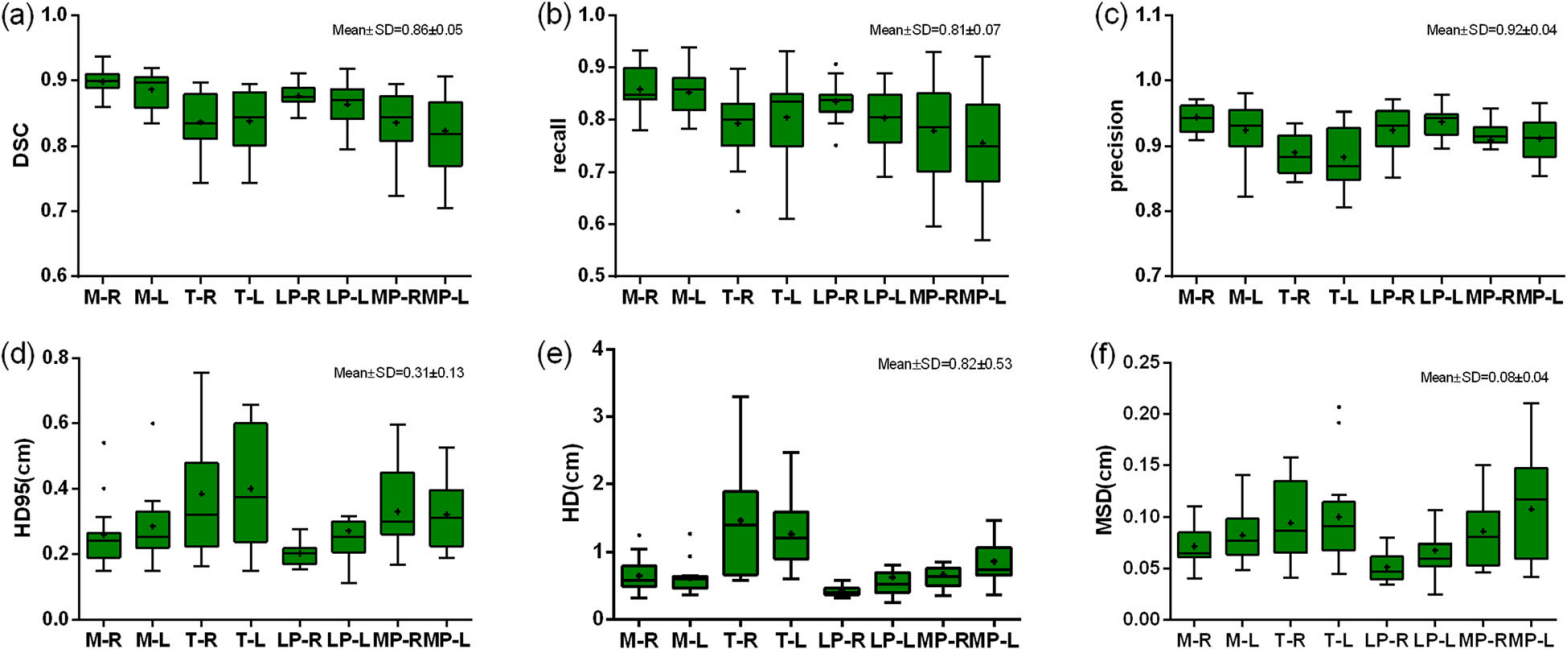
The metrics of geometric and spatial similarity for all muscles manually delineated by three clinicians (interobserver variation). In each box, the central mark is the median and edges are the 25 and 75th percentiles. and the upper and lower whiskers represents the highest and lowest values. The overall values (mean ± SD) for every metric were presented on the right upper corner for each subfigure. “+” in the box represents the mean values

Figure 2 shows metrics of geometric and spatial similarity for all the structures manually delineated by the three clinicians. Overall, both T and MP were associated with lower values for DSC, recall, and precision compared with M and LP. Higher values for MSD and HD95/HD were observed for T and MP. Among all structures, T had the highest HD95/HD. More specifically, the mean value of DSC for M, T, LP, MP ranged from 0.82 ± 0.06 to 0.90 ± 0.02, with an overall mean of 0.86 ± 0.05. The mean value ranges of HD and HD95 were from 0.42 ± 0.08 to 1.46 ± 0.85 and from 0.20 ± 0.03 to 0.40 ± 0.17, respectively, with overall means of 0.82 ± 0.53 and 0.31 ± 0.13 (unit: cm). The mean values of MSD ranged from 0.05 ± 0.01 to 0.11 ± 0.05, with an overall mean of 0.08 ± 0.04 (unit: cm). The overall means of six metrics are shown in each sub-figure, which were used as the reference values for calculating scores.

Table 2 summarizes DLAS and ABAS geometrics indices for MM segmentations. DLAS was superior to ABAS for all quantitative metrics. More specifically, DSC was 0.86 ± 0.03 and 0.83 ± 0.04 for DLAS and ABAS, respectively, as compared to the inter-observer variation baseline of 0.86 ± 0.05. HD95 was 0.30 ± 0.09 for DLAS and 0.37 ± 0.13 for ABAS, as compared to the baseline 0.31 ± 0.13. MSD was 0.08 ± 0.02, 0.11 ± 0.03, 0.08 ± 0.04 for DLAS, ABAS, and baseline, respectively. Overall, DLAS achieved equivalent performance compared to the mean interobserver variation for quantitative metrics, with smaller standard deviation (SD), except for precision. These results demonstrate that DLAS is more geometrically accurate and reproducible compared to ABAS, and the comparison showed statistical significance (*p* < 0.05) for all metrics except for precision.

**Table 2:** Mean values and standard deviation (Mean ± SD) for the 6 metrics across all organs contoured using three methods: A. DLAS; B. ABAS; C. interobserver variation (baseline)

Metrics	DLAS	ABAS	interobserver variation	*P* value*		
				A vs B	A vs C	B vs C
DSC	0.86 ± 0.03	0.83 ± 0.04	0.86 ± 0.05	0.00	0.26	0.00
Recall	0.86 ± 0.05	0.81 ± 0.07	0.81 ± 0.07	0.00	0.00	0.91
Precision	0.85 ± 0.05	0.85 ± 0.07	0.92 ± 0.04	0.97	0.00	0.00
HD95	0.30 ± 0.09	0.37 ± 0.13	0.31 ± 0.13	0.00	0.20	0.00
HD	0.73 ± 0.31	0.83 ± 0.37	0.82 ± 0.53	0.00	0.84	0.03
MSD	0.08 ± 0.02	0.11 ± 0.03	0.08 ± 0.04	0.00	0.20	0.00

* represents T test was performed among these three methods

**Fig. 3:**
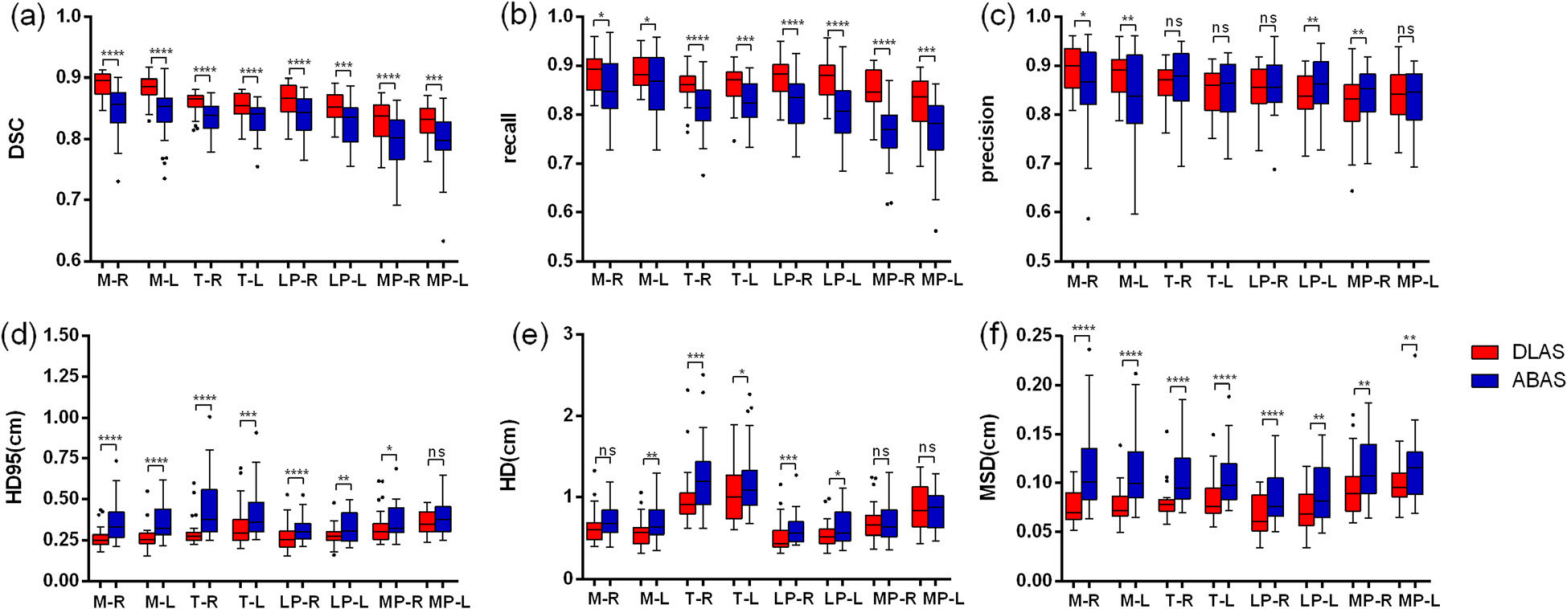
Comparison DLAS and ABAS performance. The performance was evaluated with (**a**) DSC, (**b**) recall, (**c**) precision, (**d**) HD95, (**e**) HD, (**f**) MSD. In each box, the central mark is the median and edges are the 25 and 75th percentiles and the upper and lower whiskers represents the highest and lowest values. Paired t test was used for analysis. *P < 0.05, ** *P* < 0.01, *** *P* < 0.001, **** *P* < 0.0001, ns, no significance

Figure 3 shows overall improvements in geometrics metrics for each pair MM when using DLAS, as compared to ABAS. Mean DSC for MM structures ranged from 0.79 ± 0.05 to 0.85 ± 0.04 for ABAS, and 0.83 ± 0.03 to 0.89 ± 0.02 for DLAS. When using DLAS, mean recall for all structures was also higher, while mean precision was similar with ABAS or slightly worse for some structures. For MM auto-segmentation structures, MP had the lowest DSC and recall value compared with other structures, and LP had the lowest MSD value. However, T had a larger HD/HD95 value compared with other structures. This can be explained by the larger volume of T muscles. Except for precision, paired t-test indicated that DLAS performed better than ABAS for all the metrics of each MM structure with statistically significance (*p* < 0.05).

**Fig. 4:**
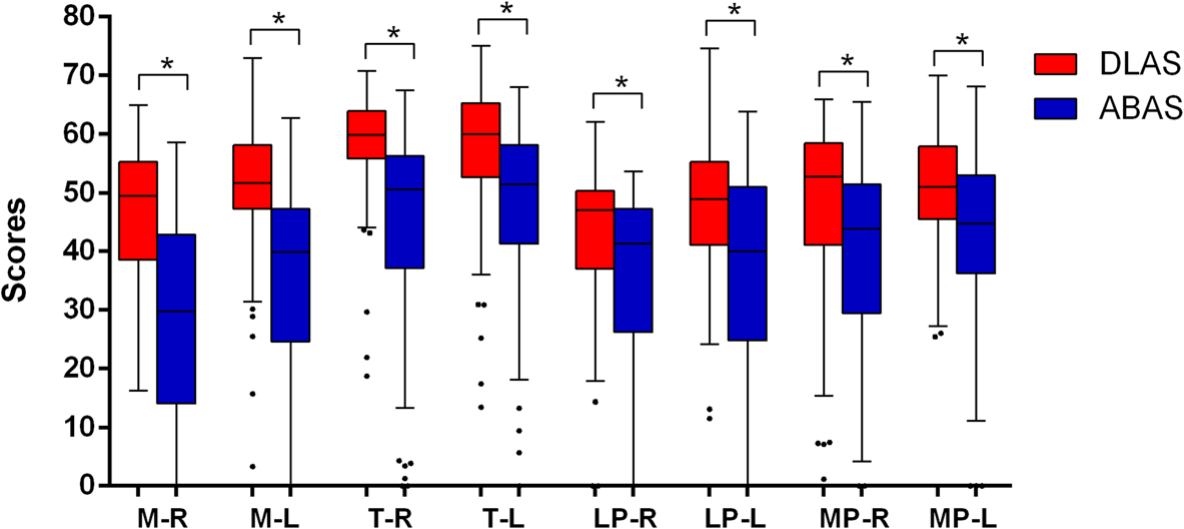
The overall scores achieved by DLAS and ABAS for all pairs of muscles. *P < 0.05. In each box, the central mark is the median and edges are the 25 and 75th percentiles and the upper and lower whiskers represents the highest and lowest values

The overall scores achieved by the two methods for every muscle is summarized in Fig. 4. The highest scores were achieved for T by both methods. For most muscle pairs, DLAS-generated structures had mean scores above 50 while ABAS was less than 50, all with statistical significance (*p* < 0.05), which indicates ABAS is inferior to the reference established based on the inter-observer variation.

Table 3 shows the percentages (%) of cases where auto-segmentation performed worse than manual segmentation by compared with the mean DSC of inter-observer variation for each muscle. The percentages of cases that performed worse than manual segmentation ranged from 20.7 to 65.5% for DLAS, and from 41.4 to 96.6% for ABAS. Chi-Square test showed that the difference was statistically significant for most of the structures(*p <* 0.05). These results indicate that DLAS performance is superior compared to ABAS and that ABAS segmentations require more contour revision to achieve equivalence. Among all MMs, T segmentations with either DLAS or ABAS had the fewest number of cases performing worse than that of manual segmentations.

**Table 3:** The percentages (%) of cases for each muscle auto segmented by DLAS and ABAS which were worse than that achieved by physicians (mean DSC was used to compare the results)

	M-R	M-L	T-R	T-L	LP-R	LP-L	MP-R	MP-L
DLAS	62.1%	51.7%	20.7%	24.1%	65.5%	65.5%	44.8%	37.9%
	(18/29)	(15/29)	(6/29)	(7/29)	(19/29)	(19/29)	(13/29)	(11/29)
ABAS	96.6%	89.7%	48.3%	41.4%	96.6%	82.8%	79.3%	69.0%
	(28/29)	(26/29)	(14/29)	(12/29)	(28/29)	(24/29)	(23/29)	(20/29)
*P* value*	0.02	0.03	0.05	0.26	0.01	0.23	0.01	0.03

* represents Chi-Square test was performed between DLAS and ABAS

### Dosimetric impact of variation in contouring

**Fig. 5:**
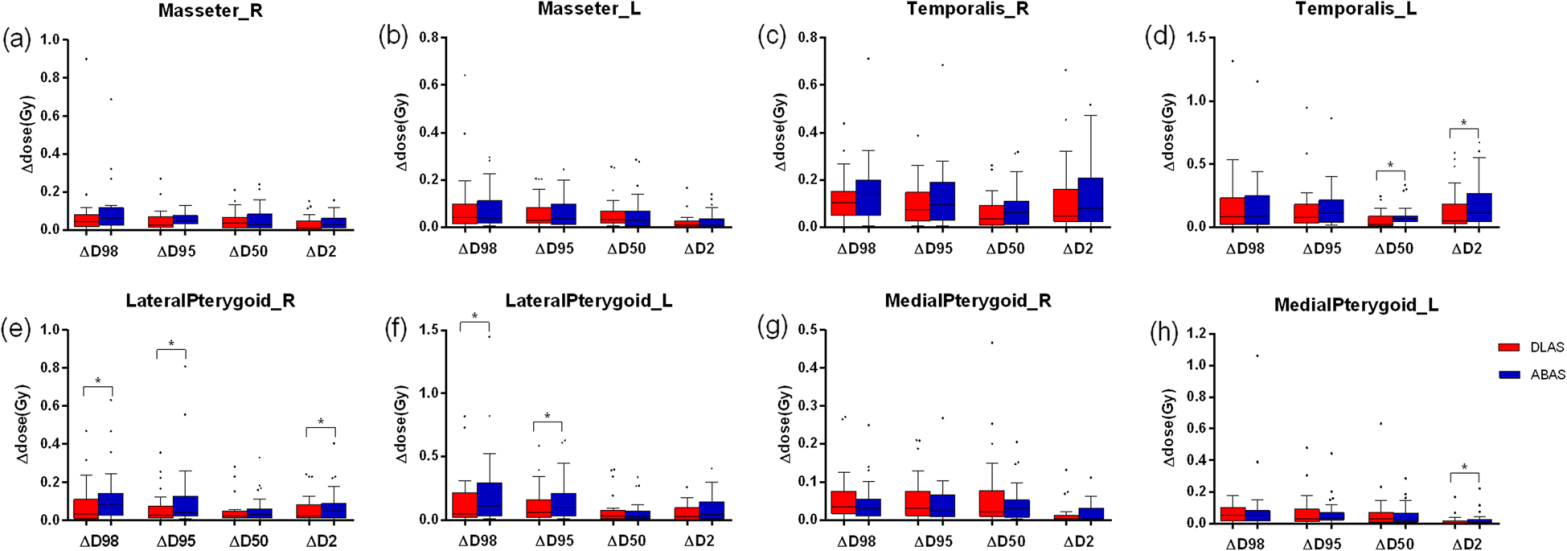
Comparisons of ∆dose of DLAS vs ABAS. Paired t test was used for analysis. *P < 0.05. In each box, the central mark is the median and edges are the 25 and 75th percentiles. and the upper and lower whiskers represents the highest and lowest values

Figure 5 shows dosimetric endpoints for DLAS and ABAS segmentations for paired MMs. Box plots show ∆dose of each muscle for DLAS and ABAS. The mean ∆D98%, ∆D95%, ∆D50%, and ∆D2% for most of the structures was less than 10%. However, ∆D98% and ∆D95% were large in some cases, such as ∆D98% of T-L, LP-L, MP-L for three cases was up to 100%. In addition, one case showed ∆D50% of MP-L was more than 50% (absolute dose greater than 10Gy). Among these cases, ipsilateral MMs showed larger degrees of dose variation compared with the contralateral muscles. These findings indicate that, for the organs in a steep dose gradient, segmentation variability of several millimeters may drastically change MM dosimetric endpoints. Comparison of ∆dose for DLAS and ABAS revealed generally similar results, the difference was not statistically significant for most of the cases (*P* > 0.05). However, dose to MMs with DLAS more closely matched manual segmentations than did ABAS.

## Discussion

This is the first study to assess the feasibility of a deep learning method for contouring masticatory muscles in head and neck radiotherapy. Results indicate that our in-house DLAS as compared to the commercial ABAS tool provides accurate, consistent, reproducible MM counters without the need of any manual correction or user interference. Dosimetric comparison of MMs for DLAS and ABAS shows that the dose difference from that of manual contours has a minimal clinical impact with less variation and improved consistency.

Many studies [6, 28, 34, 35] had characterized inter-observer variation in contouring. Yang et al .[28] used three cases by three observers to measure inter-rater variability in thoracic OAR segmentation. Nelms et al. [6] provided one patient CT data to several physicians to quantify the OAR contouring variation in the head and neck. We selected five cases and three physicians to estimate the interobserver variation for the MM contouring. While a larger dataset, or more observers, may help improve the statistical power of analysis. The focus of this study is not an accurate measurement of interobserver variability, but rather to provide a rough reference when evaluating the automatic algorithms’ performance. Our results suggest that there is contour variation between observers. Among all the structures, T and MP showed more variation indicating that T and MP are more difficult to define anatomically. Prior to clinical implementation it is important to determine if these automated segmentation results fall within the variability seen with manual segmentation. Comparison of quantitative geometric indices showed that DLAS of MMs was more reproducible (less variable) than manual segmentations. The results of overall scores also indicates that DLAS perform better than ABAS, and DLAS segmentations require less contour revision before using clinically.

Several studies [7–9, 17, 18, 36] previously evaluated the performance of different methods of auto-segmentation for head and neck radiotherapy. Hague *et al* [18] developed a new contouring atlas to evaluate the reduction in interobserver variability for MP, LP, M, and T muscles. The authors found that an atlas reduced interobserver variability for all muscles and the mean DTA improved when the trainees used the atlas. Furthermore, they found that T had the largest reduction in variability (4.3 ± 7.1 v1.2 ± 0.4 mm, *p* = 0.06), and for MP and T the distance between the center of mass (COM) and interobserver variability reduced in all directions. Our results indicated that DLAS was associated with smaller contour variation for all muscles compared to ABAS, with a higher mean DSC, Recall and a lower mean HD/HD95 and MSD, while precision stayed on a similar level. It means that DLAS has increased overlap with the ground-truth contours without over-contouring.

Comparison of MM segmentation strategy (DLAS, ABAS, manual segmentation) showed small contouring differences (on the order of millimeters) in general. While the dosimetric impact for those contouring differences was usually small, large dosimetric differences did occur. In this study, we found there is one case for which ∆D50% of MP-L was up to 50% (≥10Gy in absolute dose). A closer inspection showed that the structure passed through the penumbra region created by the jaw. The dose gradient for the jaw penumbra was >4Gy/mm. Thus, geometric errors on the order of 2.5 mm produced a > 10Gy change in absolute dose. This indicates that segmentation accuracy in areas with high dose and steep dose gradients is important.

So far, there are a few studies investigating the dose-volume factors correlating with trismus. Molen et al. [25]. found that dose-parameters (mean, max, V20, V40, and V60) of all mastication structures had strong correlation with subjective mouth-opening problems at 1-year. It was also observed that [37] after a dose of 40 Gy, the probability of trismus will increase 24% for every 10 Gy in the pterygoid muscle. If trismus-related muscles were irradiated bilaterally [37], it will also increase the incidence of trismus. Other authors [22, 24, 38] indicated that mean radiation dose to the ipsilateral structures (i.e. masseter and medial pterygoid muscles) is an important risk factor. While small dose differences were observed for most cases in our study, depending on the location of the tumor and high dose gradient location, MMs can receive high doses and should be given consideration during the planning process.

This study validated a deep learning model for fast auto-segmentation of the MMs. Using the DLAS, there was a reduction in variability of contours of all muscles. It also increases clinical efficiency in eliminating manual contouring time. This method should be easily adopted by other radiotherapy centers to improve structure delineation consistency for head and neck patients, which may also help to aid the development consistency in multi-institutional clinical trials.

There are potential limitations in our study. The “ground truth” contours are based on manual contours created by the physician. The contouring bias of the physician may impact our results. However, we adopted strategies to minimize this bias. Contouring guidelines of the published study by Rao et al. [22] were followed. In addition, all manual contours were reviewed carefully by an expert before they were used in this research. Another limitation is the limited dataset size. Twenty-seven cases were used for training and twenty-nine cases were used for testing. It is possible that our results are biased due to the limited variety of cases and possible imbalance of case representations in training and testing dataset. In addition, while the performance for both DLAS and ABAS would improve with more training data, it is possible that one method may benefit more than the other. For future studies, we plan to create a larger dataset and compare the performance gain of both methods as training data increases.

## Conclusion

In summary, a deep learning model was validated for the automatic segmentation of the mastication muscles for improving workflow and efficiency in the radiation therapy treatment planning process. This method has been shown to significantly improve consistency in contouring of all masticatory muscles compared with a commercial ABAS method, or manual segmentation. It is important to note that this study identifies the importance of contouring and dose monitoring for well lateralized oral cavity or oropharyngeal tumors during the planning phases, in which segmentation variability of several millimeters may drastically change ipsilateral MMs dosimetric endpoints.

## Data Availability

The datasets used and/or analyzed during the current study are available from the corresponding author on reasonable request.

## Abbreviations

CT: Computed tomography
M: Masseter
T: Temporalis
MP: Medial pterygoid
LP: Lateral pterygoid
MMs: Masticatory muscles
DLAS: Deep learning auto-segmentation
ABAS: Atlas-based auto-segmentation
DSC: Dice similarity coefficient
HD: Hausdorff distance
MSD: Mean surface distance
OARs: Organs at risk
HNC: Head and neck cancer

## References

[1] Mackie TR, Kapatoes J, Ruchala K (2003). Image guidance for precise conformal radiotherapy. Int J Radiat Oncol Biol Phys.

[2] Gomez-Millan J, Fernandez JR, Medina Carmona JA (2013). Current status of IMRT in head and neck cancer. Rep Pract Oncol Radiother.

[3] Brouwer CL, Steenbakkers RJ, van den Heuvel E (2012). 3D variation in delineation of head and neck organs at risk. Radiat Oncol.

[4] Peng YL, Chen L, Shen GZ (2018). Interobserver variations in the delineation of target volumes and organs at risk and their impact on dose distribution in intensity-modulated radiation therapy for nasopharyngeal carcinoma. Oral Oncol.

[5] Moore A (2018). Observer variation in the delineation of organs at risk for head and neck radiation therapy treatment planning: a systematic review protocol. JBI Database System Rev Implement Rep.

[6] Nelms BE, Tome WA, Robinson G (2012). Variations in the contouring of organs at risk: test case from a patient with oropharyngeal cancer. Int J Radiat Oncol Biol Phys.

[7] Daisne JF, Blumhofer A (2013). Atlas-based automatic segmentation of head and neck organs at risk and nodal target volumes: a clinical validation. Radiat Oncol.

[8] Yang J, Beadle BM, Garden AS (2014). Auto-segmentation of low-risk clinical target volume for head and neck radiation therapy. Pract Radiat Oncol.

[9] Qazi AA, Pekar V, Kim J (2011). Auto-segmentation of normal and target structures in head and neck CT images: a feature-driven model-based approach. Med Phys.

[10] Dean JA, Welsh LC, McQuaid D (2016). Assessment of fully-automated atlas-based segmentation of novel oral mucosal surface organ-at-risk. Radiother Oncol.

[11] Kieselmann JP, Kamerling CP, Burgos N (2018). Geometric and dosimetric evaluations of atlas-based segmentation methods of MR images in the head and neck region. Phys Med Biol.

[12] Lin L, Dou Q, Jin YM (2019). Deep learning for automated contouring of primary tumor volumes by MRI for nasopharyngeal carcinoma. Radiology.

[13] Isambert A, Dhermain F, Bidault F (2008). Evaluation of an atlas-based automatic segmentation software for the delineation of brain organs at risk in a radiation therapy clinical context. Radiother Oncol.

[14] Hoang Duc AK, Eminowicz G, Mendes R (2015). Validation of clinical acceptability of an atlas-based segmentation algorithm for the delineation of organs at risk in head and neck cancer. Med Phys.

[15] Zhu W, Huang Y, Zeng L (2019). AnatomyNet: deep learning for fast and fully automated whole-volume segmentation of head and neck anatomy. Med Phys.

[16] Ibragimov B, Xing L (2017). Segmentation of organs-at-risks in head and neck CT images using convolutional neural networks. Med Phys.

[17] Teguh DN, Levendag PC, Voet PW (2011). Clinical validation of atlas-based auto-segmentation of multiple target volumes and normal tissue (swallowing/mastication) structures in the head and neck. Int J Radiat Oncol Biol Phys.

[18] Hague C, Beasley W, Dixon L (2019). Use of a novel atlas for muscles of mastication to reduce inter observer variability in head and neck radiotherapy contouring. Radiother Oncol.

[19] Weber C, Dommerich S, Pau HW (2010). Limited mouth opening after primary therapy of head and neck cancer. Oral Maxillofac Surg.

[20] Scott B, Butterworth C, Lowe D (2008). Factors associated with restricted mouth opening and its relationship to health-related quality of life in patients attending a maxillofacial oncology clinic. Oral Oncol.

[21] Louise Kent M, Brennan MT, Noll JL (2008). Radiation-induced trismus in head and neck cancer patients. Support Care Cancer.

[22] Rao SD, Saleh ZH, Setton J (2016). Dose-volume factors correlating with trismus following chemoradiation for head and neck cancer. Acta Oncol.

[23] Pauli N, Johnson J, Finizia C (2013). The incidence of trismus and long-term impact on health-related quality of life in patients with head and neck cancer. Acta Oncol.

[24] Gebre-Medhin M, Haghanegi M, Robert L (2016). Dose-volume analysis of radiation-induced trismus in head and neck cancer patients. Acta Oncol.

[25] van der Molen L, Heemsbergen WD, de Jong R (2013). Dysphagia and trismus after concomitant chemo-intensity-modulated radiation therapy (chemo-IMRT) in advanced head and neck cancer; dose-effect relationships for swallowing and mastication structures. Radiother Oncol.

[26] Jatin P (2018). Shah PHM: New AJCC/UICC staging system for head and neck,and thyroid cancer. Rev Med Clin Condes.

[27] Çiçek Ö, Abdulkadir A, Lienkamp SS, et al. 3D U-net: learning dense volumetric segmentation from sparse annotation, International Conference on Medical Image Computing and Computer-Assisted Intervention: Springer; 2016. p. 424–32.

[28] Yang J, Veeraraghavan H, Armato SG (2018). Autosegmentation for thoracic radiation treatment planning: a grand challenge at AAPM 2017. Med Phys.

[29] Cardenas CE, Mohamed AS, Yang J (2020). Head and neck cancer patient images for determining auto-segmentation accuracy in T2-weighted magnetic resonance imaging through expert manual segmentations. Med Phys.

[30] Feng X, Bernard ME, Hunter T, et al. Improving accuracy and robustness of deep convolutional neural network based thoracic OAR segmentation. Phys Med Biol. 2020. 10.1088/1361-6560/ab7877 PMC8035811 32079002

[31] Feng X, Qing K, Tustison NJ, et al. Deep convolutional neural network for segmentation of thoracic organs-at-risk using cropped 3D images. Med Phys. 2019. 10.1002/mp.13466 30830685

[32] Delpon G, Escande A, Ruef T (2016). Comparison of automated atlas-based segmentation software for postoperative prostate Cancer radiotherapy. Front Oncol.

[33] Weistrand O, Svensson S (2015). The ANACONDA algorithm for deformable image registration in radiotherapy. Med Phys.

[34] Fiorino C, Reni M, Bolognesi A (1998). Intra- and inter-observer variability in contouring prostate and seminal vesicles: implications for conformal treatment planning. Radiother Oncol.

[35] Foroudi F, Haworth A, Pangehel A (2009). Inter-observer variability of clinical target volume delineation for bladder cancer using CT and cone beam CT. J Med Imaging Radiat Oncol.

[36] Lee H, Lee E, Kim N (2019). Clinical evaluation of commercial atlas-based auto-segmentation in the head and neck region. Front Oncol.

[37] Teguh DN, Levendag PC, Voet P (2008). Trismus in patients with oropharyngeal cancer: relationship with dose in structures of mastication apparatus. Head Neck.

[38] Lindblom U, Garskog O, Kjellen E (2014). Radiation-induced trismus in the ARTSCAN head and neck trial. Acta Oncol.

